# Sleep and Its Disorders.—II

**Published:** 1896-01-18

**Authors:** Tom R. Taylor


### MEDICAL PROGRESS AND HOSPITAL CLINICS.

[*The Editor will be glad to receive offers of co-operation and contributions from members of the profession. All letters should be addressed to* THE EDITOR, AT THE OFFICE, 428, STRAND, LONDON, W.C.]

### SLEEP AND ITS DISORDERS.—II.

By TOM R. TAYLOR, M.D., B.S., B.Sc.Lond., F.R.C.S.Eng.

**Prolonged Sleep.**—Apart from the conditions of trance or catalepsy and the artificially-induced sleep of hypnotism, we hear but little of excessive sleep. More or less demented individuals, however, pass long periods in a state of stuporose slumber.

The heaviness and sleepiness complained of by the majority of those who consult us are almost invariably the result of a tco great love for "the flesh pots of Egypt." In nine cases out of ten it means chronic dyspepsia and constipation. An uncongenial occupation or excessive work will account for nearly all the rest.

On the west coast of Africa there is a curious endemic disease known as "the sleeping sickness." The patient becomes gradually more and more somnolent, and finally passes into a state of coma, and, as a rule, dies. It must be remembered in connection with excessive sleepiness, that it often occurs in cases of grave toxæmia, and Friedreich and Obernier record two cases of cerebral tumour where it was the chief symptom.

**Dreams.**—Locke says: "The mind can frame unto itself no one new simple idea." It requires a wide education, a retentive memory, and a brilliant imagination for a man to realise dreams such as those of De Quincey. Not everyone who drugs himself with laudanum experiences the intellectual heaven described by "the opium eater." Dreams are but the resuscitation of apparently dead memories. Impressions never entirely fade from the mind. The hemispheres act as it were as store-houses. But experiences do not remain for ever, or even long in their original condition, and the memory called up by some associated idea is often unrecognisable. In sound sleep there are no dreams, and it appears from the scanty scientific knowledge we have of them that they occur either at the time when we are falling asleep or waking—a condition of partial sleep. The higher functions of judgment, comparison, and will are obliterated, and with them the power of perception of time and space. The senses are active to a certain extent, and it is through them that the initial process in a dream is started. Habitual, recent, and any extremely interesting impressions are then recalled.

They may be vivid and detailed, and the experience bear all the appearance of the past event being re-enacted; or the incidents, details, and mere mental feelings of several memories may be blended into an apparently new creation. On the other hand, no concrete conception may be formed, and the whole is but a confused medley of disjointed ideas. The term micro-psychical may be applied to these. A dream may occur time after time with exactly the same feelings attached to it. Many students have a special dream before every examination, and similarly with those in other walks of life.

am acquainted with a man who became quite dejected as to the results of an enterprise if he did not have a certain peculiar dream. From time immemorial visions have been viewed with superstitious awe, and even at the present time we tell our dreams, and often find a quasi-prophet who interprets. Living, as it were, another life, where events apparently new take place, where the whole interest of the drama centres upon what in waking life would be perhaps ignored, it naturally follows that a mystic importance is attached to the idea upon which the dream is founded. An explanation once wanted can always be found, and in this particular case the supply exceeds the demand. It is amusing and interesting to note the varying interpretations given by different authorities. If a rhyming couplet be the form of the oracular opinion, then so much the more does the dictum carry weight. For example, take the case of dreaming of fruit—

"Fruit out of season,
Pain without reason."

This opinion is objected to by another prophet, who says, to dream of fruit portends success and happiness. The former, however, is the one most relied upon, and in the present order of things, the most generally true.

In dreams, perhaps, we most distinctly realise the fact that pleasure and pain are feelings absolutely distinct from the objects or sensations which evoke them. We may be absurdly happy or suicidally miserable over exactly the same idea. A very remarkable fact is the effect of a miserable dream upon the spirits for some time, even days, after it has passed away, even upon the least superstitious of mortals. Nightmare or night terrors are an absolute affliction to many people, and children suffer as much or more than adults. They are often of a recurrent type, the same horror occurring over and over again.

A friend relates how as a young child he was haunted in his sleep night after night by a dream of a corpse. He saw the vision with agonising terror. The horror moved to clutch him, and he awoke with a shriek. He had at that time never seen a dead body. At first sight it seems difficult to realize the processes at work. But we must note that the idea of death was supplied gratuitously, for the hallucination was a moving one. All or most young children have heard of death, and unconsciously, in fact, naturally, acquire a horror of all connected with it. Add to an ordinary man the idea of death, absolute dread, and a sense stimulus as causative agent, and we have all the ingredients required for this apparent contradiction to the statement that dreams are old ideas resuscitated.

All researches upon the subject tend to show that the most detailed and wonderfully intricate dramas are played in dreamland in perhaps a few seconds. The story related of Mohammed, who in the time it took for a basin to fall from a table to the ground dreamed a whole life's history, is only a slight exaggeration.

**Somnambulism**, often a most troublesome and dangerous habit, is closely allied to the above condition.

It takes many forms, but in all cases must be regarded as a variety of partial sleep. The commonest form is that in which the victim actually acts his part in a dream and has some recollection afterwards. Once when working hard at mathematics and much troubled about a certain problem, I started out of bed in such a condition, and actually commenced wandering about the house "to find the solution." It is a remarkable fact that somnambulists are almost invariably attracted to a window or an open door. This must certainly be accounted for on the supposition that there is some perception of light. This peculiarity has ended in a tragic manner in many cases, and countless stories tell of hairbreadth escapes and feats performed impossible to the waking individual. Somnambulistic performances during a more profound degree of mental sleep, where no memory remains, are closely akin to the phenomena of hypnotism, and must also be ascribed to obsession or the predominance of a certain fixed idea. In my own case mentioned above there was a certain idea that something must be found, and forthwith when the higher centres were asleep the suggestion was paramount.

**Somniloquy**, or sleep-talking, may occur either together with the last condition or as a phenomenon unassociated with any other motor manifestations. A coherent conversation may be carried on with another real or imaginary person, or the remarks may be limited to disjointed sentences or even single words. Nearly everyone talks, or has talked, during sleep, but few either see or hear of a case such as that recorded by Dr. Bastian in Quain's "Dictionary of Medicine." A young lady sang during sleep in a manner impossible for her to do when awake. One is involuntarily compelled to think of the hypnotised Trilby. One of the boys in the same dormitory as myself at school once aroused the rest by raising an alarm of fire. We were on the alert on once. He answered quite coherently, and described the advancing fire, shrieking with terror. He was quite oblivious to his real surroundings until rudely brought back to the workaday world by a missile hurled by a fierce and sleepy neighbour.

In both somnambulism and somniloquy, parts only of the brain seem to be asleep, and those allotted to certain functions appear not only to be awake, but more than usually receptive of certain stimuli. The condition is closely akin to the condition known to hypnotists as the agitated variety of the somnambulistic stage. And as in this latter state the subject sees and hears only what is suggested, so, in the natural form, only those impressions are perceived which are consistent with the dream.

**Treatment.**—With regard to the treatment of these forms of partial sleep, what are we to say?

Some authorities go so far as to say that no sleep is absolutely dreamless, but that after apparently sound sleep no memory remains. There is no doubt that many of the most awful forms of nightmare, with the resulting exhaustion and mental depression, are purely bodily in origin, and may be accounted for by late or heavy suppers. On the other hand, there is generally some neurotic taint in those who indulge in somnambulism and somniloquy. These are by far the most difficult cases to manage. The traditional treatment of a "sousing" in cold water is, at any rate, not to be employed for sleep-walkers.

Our lines of treatment must be, as far as possible, purely hygienic diet, exercise, and fresh air, in order to make sleep as sound as possible. In the more complicated cases it may be necessary to give sedatives, and then the surest, and by far the best, remedy is a combination of bromides with ammonia. The mental side of the patient must not be neglected, and as healthy a tone as possible must be cultivated and encouraged. Anxiety, worry, and over-work must be reduced to a minimum, and any fixed idea or trouble as far as possible alleviated by a change of scene and surroundings.

